# A Needs Assessment for a Longitudinal Emergency Medicine Intern Curriculum

**DOI:** 10.5811/westjem.2016.9.31493

**Published:** 2016-11-08

**Authors:** Eric Shappell, James Ahn

**Affiliations:** University of Chicago, Department of Medicine, Section of Emergency Medicine, Chicago, Illinois

## Abstract

**Introduction:**

A key task of emergency medicine (EM) training programs is to develop a consistent knowledge of core content in recruits with heterogeneous training backgrounds. The traditional model for delivering core content is lecture-based weekly conference; however, a growing body of literature finds this format less effective and less appealing than alternatives. We sought to address this challenge by conducting a needs assessment for a longitudinal intern curriculum for millennial learners.

**Methods:**

We surveyed all residents from the six EM programs in the greater Chicago area regarding the concept, format, and scope of a longitudinal intern curriculum.

**Results:**

We received 153 responses from the 300 residents surveyed (51% response rate). The majority of respondents (80%; 82% of interns) agreed or strongly agreed that a dedicated intern curriculum would add value to residency education. The most positively rated teaching method was simulation sessions (91% positive responses), followed by dedicated weekly conference time (75% positive responses) and dedicated asynchronous resources (71% positive responses). Less than half of respondents (47%; 26% of interns) supported use of textbook readings in the curriculum.

**Conclusion:**

There is strong learner interest in a longitudinal intern curriculum. This needs assessment can serve to inform the development of a universal intern curriculum targeting the millennial generation.

## INTRODUCTION

A key task of emergency medicine (EM) training programs is to develop a consistent knowledge of core content in recruits with heterogeneous training backgrounds and variable gaps in education.^1^ The traditional model for delivering core content is lecture-based weekly conference; however, a growing body of literature finds this format less effective and less appealing than alternatives.^2^–^6^ As a result, some training programs have introduced new teaching methods such as shorter and more interactive lectures, small group sessions, and web-based asynchronous components.^7^,^8^ These advances herald the adaptation of conference design to meet the challenges of educating today’s millennial learners who have “little desire to read long texts,”^9^ value appropriate usage of technology, and seek interactive learning opportunities.^10^,^11^

Compounding the challenge of engaging millennials, the traditional model of delivering the same content to all training levels has limited educational return.^12^ Topics appropriate for interns are unlikely to be high yield for senior residents, whereas advanced topics can be inappropriate for novice learners. Finally, the traditional model may suffer from a limited audience during weekly conference; it is not uncommon for residents to miss conference due to clinical or other obligations.^13^

We sought to address these challenges by developing a novel longitudinal intern curriculum for millennial learners using the framework of Kern’s six-step model for curriculum development.^14^ The first steps in this model are problem identification and a targeted needs assessment. Within this framework, the problem was the absence of a longitudinal curriculum tailored for interns that was consistently available. We conducted a literature review that revealed two examples of intern boot camp development.^15^,^16^ However, we were unable to identify a longitudinal curriculum for intern-level learners, and there are no published reports of a needs assessment for an EM intern-level curriculum. We aim to fill this gap in the literature by conducting a targeted needs assessment of EM residents on the concept, format, and scope of a longitudinal intern curriculum.

## METHODS

We surveyed all residents from the six EM programs in the greater Chicago area during the 2015–2016 academic year. These programs include two four-year training programs and four three-year training programs. Three are university-affiliated programs, two are community programs, and one is a county program.

The survey questions were developed iteratively by a working group of EM education experts with the goal of assessing resident attitudes toward the concept, format, and topics covered in a longitudinal intern curriculum (Appendix A). Example topics were chosen from core content representing three categories: emergent conditions, common complaints, and procedures/skills. We piloted the survey among a representative audience (17 residents at one institution), and established response process validity by reviewing feedback from the pilot, which resulted in the addition of six additional example topics. This survey contained both multiple-choice and free-text items. Surveys were distributed to residents via email by their respective program leadership and participation was voluntary. The survey spanned from November 2015 to April 2016. Two follow-up emails were sent (February and April of 2016) prior to closing the survey.

In order to characterize potential differences in opinion between intern respondents and the study group as a whole, we analyzed intern responses separately for questions regarding the perceived value of an intern curriculum and preferred teaching methods. Positive responses were defined as responses of “Agree” or “Strongly Agree.” Unanswered questions were treated as null. We compiled and analyzed data using Microsoft Excel (Microsoft, Redmond, WA).

## RESULTS

We received 153 responses from the 300 residents surveyed (51% response rate). Of these responses were 58 (38%) interns, 40 (26%) second-year residents, 43 (28%) third-year residents and 12 (8%) fourth-year residents. The average number of residents responding from each program was 26 ± 5.9.

Resident impressions of the educational value and preferred teaching methods in a longitudinal intern curriculum are depicted in Figures A and B, respectively. The majority of respondents (80%; 82% of interns) agreed or strongly agreed that a dedicated intern curriculum would add value to residency education. The most positively rated teaching method was simulation sessions (positive responses: 91% of all residents; 91% of interns) followed by dedicated weekly conference time (positive responses: 75% of all residents; 84% of interns) and dedicated asynchronous resources (positive responses: 71% of all residents; 69% of interns). Less than half of respondents (47%; 26% of interns) supported use of textbook readings in the curriculum. When asked how many hours of weekly conference time should be dedicated to an intern curriculum, the majority responded one hour (n = 100, 65%) followed by two hours (n = 39, 25%), no time (n = 8, 5%) and three or more hours (n = 6, 4%).

Resident opinions on suggested topics to include in a longitudinal intern curriculum are illustrated in Figure C. All potential suggested topics surveyed received over 80% positive responses other than the topics of arterial line placement (63%) and thoracotomy (43%). Topics in the open-ended portion of the survey that were submitted by more than one resident included the following: dermatology and ultrasound (three responses each), documentation, orthopedics, and toxicology (two responses each).

## DISCUSSION

This needs assessment illustrates a strong learner interest in a dedicated longitudinal intern curriculum as the more than 80% of respondents believed this type of curriculum would add value to their education. Learners primarily desire dedicated conference time that offers a “hands-on” experience. The desires of this learner group are, unfortunately, faculty and infrastructure intensive: both dedicated conference time and simulation sessions require significant effort on behalf of the program leadership responsible for organizing the curriculum and the educators running individual sessions. However, this dataset can offer an objective measure of perceived value in these investments and can be used to focus the efforts of residency leaders interested in developing an intern curriculum.

Asynchronous resources were also favorably reviewed for inclusion in the curriculum. This finding coincides with the growing number and popularity of free open-access medical education (FOAM) resources now available online.^17^,^18^ In addition, the Accreditation Council for Graduate Medical Education now allows for up to 20% of conference time to be allotted to asynchronous learning, which underscores the legitimacy of this teaching modality. Resources such as ALiEM AIR,^19^ EM Fundamentals,^20^ and Emergency Medicine Foundations^21^ have been created with this concept explicitly in mind and are ripe for inclusion as asynchronous resources in this type of curriculum. Textbook readings were unpopular with this audience; less than half of all residents and only 26% of interns responded favorably to their inclusion in an intern curriculum. This tepid response to textbook reading is likely due to the educational needs represented by millennials. In fact, the call for increased asynchronous resources and decreased textbook usage is not surprising given recommendations for teaching this group of learners.^9^

Using these data as a guide, we can draw three conclusions: 1) Learners desire a level-specific curriculum; 2) learners desire a strong simulation experience in their intern curriculum; and 3) learners have eschewed the textbook for asynchronous resources. An ideal curriculum that maximizes learner interest could include dedicating one hour of weekly conference time to training level-specific topics. The topics covered should include those most highly recommended by learners in this study. A simulation experience (ranging from high-fidelity arrangements to oral boards-style cases) should play a significant role in these weekly intern conferences. Finally, requiring learners to review asynchronous resources prior to conference would allow for a “flipped classroom” design where more time could be dedicated to simulation, discussion, as well as higher learner satisfaction and knowledge acquisition.^22^,^23^ Using the framework of Kern, the next steps in building this curriculum would be the development of goals and objectives, educational methods, curricular implementation, learner assessment, and curriculum evaluation. Before widespread deployment of such a curriculum can be justified, pilot programs will at the very least need to establish evidence of outcome non-inferiority when compared to traditional methods.

## LIMITATIONS

This survey was conducted across a small, heterogeneous group of residency programs, including both three- and four-year programs, as well as a mix of university, community, and county settings. While this variety may speak to the generalizability of the findings, the pooled results may also wash out specific program-level attitudes and perceived strengths or weaknesses. Additionally, given our response rate, there is potential for nonresponse error, as those who chose not to respond may have different curricular needs than those who did.^24^ However, since this is a needs assessment, we felt that capturing 50% of the learners yielded important information about the educational needs of this generation’s EM interns. It is also important to note that these data represent the opinions of one group of stakeholders (i.e. learners) and such information should not be used in isolation to make curricular decisions. Additionally, while the initial problem was identified by program leadership and education experts at one institution and the survey design was informed by their assessment of learner needs, we also face coverage error as additional stakeholders (e.g. outside program leadership) were not asked about perceived needs. Future study should include this group to obtain this important perspective. Also, we did not address the preferred curricular structure for the minority of residents who indicated neutrality or negative opinions regarding this type of longitudinal intern curriculum. Further study to determine how best to approach these learners is warranted. Finally, we acknowledge that all institutions may not have the necessary resources to develop a longitudinal EM intern curriculum as described by this study. The development of a universal open-access curriculum would be beneficial to programs that lack the infrastructure to create an intern curriculum locally.

## CONCLUSION

This study shows strong learner interest in a longitudinal intern curriculum. The preferred educational methods include dedicated conference and simulation time with corresponding asynchronous resources. This needs assessment can serve to inform the development of a universal longitudinal intern curriculum targeting the millennial generation.

## Supplementary Information



## Figures and Tables

**Figure ABC:**
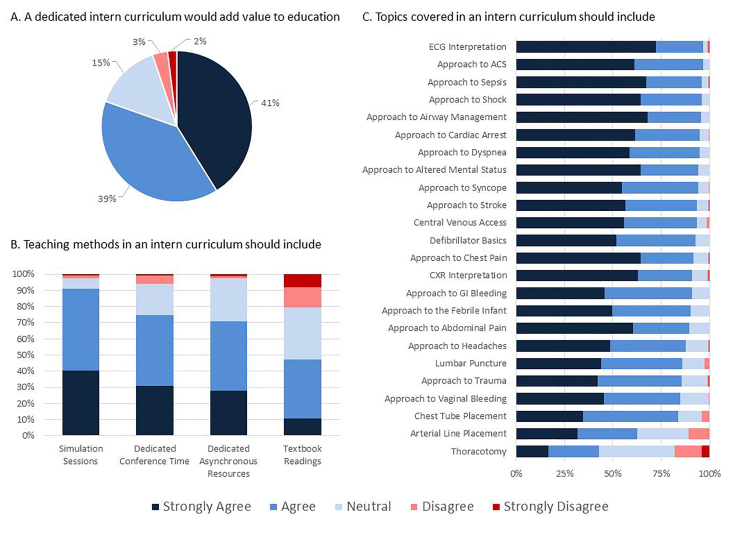
Emergency medicine resident opinions on intern curriculum value and design. *ECG*, electrocardiogram; *ACS,* acute coronary syndromes; *CXR*, chest radiograph; *GI,* gastrointestinal.
