# Supplementary material for: A Needs Assessment for a Longitudinal Emergency Medicine Intern Curriculum
Source: West J Emerg Med. 2016 Nov 8;18(1):31–4. doi: 10.5811/westjem.2016.9.31493 (PMC5226759; doi:10.5811/westjem.2016.9.31493)
Supplement: Supplementary file 1 [file wjem-18-31-s001.pdf]

I believe that a dedicated intern curriculum would add value to residency education

□ Strongly Disagree □ Disagree □ Neutral □ Agree □ Strongly Agree

An intern curriculum should involve dedicated time during weekly conferences

□ Strongly Disagree □ Disagree □ Neutral □ Agree □ Strongly Agree

How many hours of conference per week should be dedicated to an intern curriculum?

□ 0 □ 1 □ 2 □ 3+

An intern curriculum should involve dedicated simulation time

□ Strongly Disagree □ Disagree □ Neutral □ Agree □ Strongly Agree

How many hours of simulation conference per month should be dedicated to an intern curriculum?

□ 0 □ 1 □ 2 □ 3+

An intern curriculum should involve dedicated asynchronous resources

□ Strongly Disagree □ Disagree □ Neutral □ Agree □ Strongly Agree

An intern curriculum should involve textbook readings

□ Strongly Disagree □ Disagree □ Neutral □ Agree □ Strongly Agree

---

Please rate your level of agreement with the statement that each of the following is a necessary topic to be included in an intern curriculum:

Approach to Acute Coronary Syndromes

□ Strongly Disagree □ Disagree □ Neutral □ Agree □ Strongly Agree

Approach to Airway Management

□ Strongly Disagree □ Disagree □ Neutral □ Agree □ Strongly Agree

Approach to Cardiac Arrest

□ Strongly Disagree □ Disagree □ Neutral □ Agree □ Strongly Agree

Approach to Sepsis

□ Strongly Disagree □ Disagree □ Neutral □ Agree □ Strongly Agree

Approach to Shock

□ Strongly Disagree □ Disagree □ Neutral □ Agree □ Strongly Agree

Approach to Stroke

□ Strongly Disagree □ Disagree □ Neutral □ Agree □ Strongly Agree

Approach to Trauma

□ Strongly Disagree □ Disagree □ Neutral □ Agree □ Strongly Agree

Approach to Abdominal Pain

□ Strongly Disagree □ Disagree □ Neutral □ Agree □ Strongly Agree

Approach to Altered Mental Status

□ Strongly Disagree □ Disagree □ Neutral □ Agree □ Strongly Agree

Approach to Chest Pain

□ Strongly Disagree □ Disagree □ Neutral □ Agree □ Strongly Agree

Approach to Dyspnea

□ Strongly Disagree □ Disagree □ Neutral □ Agree □ Strongly Agree

Approach to the Febrile Infant

□ Strongly Disagree □ Disagree □ Neutral □ Agree □ Strongly Agree

Approach to GI Bleeding

□ Strongly Disagree □ Disagree □ Neutral □ Agree □ Strongly Agree

Approach to Headaches

□ Strongly Disagree □ Disagree □ Neutral □ Agree □ Strongly Agree

Approach to Syncope

□ Strongly Disagree □ Disagree □ Neutral □ Agree □ Strongly Agree

Approach to Vaginal Bleeding

□ Strongly Disagree □ Disagree □ Neutral □ Agree □ Strongly Agree

Arterial Line Placement

□ Strongly Disagree □ Disagree □ Neutral □ Agree □ Strongly Agree

Central Venous Access

□ Strongly Disagree □ Disagree □ Neutral □ Agree □ Strongly Agree

Chest Tube Placement

□ Strongly Disagree □ Disagree □ Neutral □ Agree □ Strongly Agree

Thoracotomy

□ Strongly Disagree □ Disagree □ Neutral □ Agree □ Strongly Agree

CXR Interpretation

□ Strongly Disagree □ Disagree □ Neutral □ Agree □ Strongly Agree

ECG Interpretation

□ Strongly Disagree □ Disagree □ Neutral □ Agree □ Strongly Agree

Defibrillator Basics

□ Strongly Disagree □ Disagree □ Neutral □ Agree □ Strongly Agree

Lumbar Puncture

□ Strongly Disagree □ Disagree □ Neutral □ Agree □ Strongly Agree

---

Other topics that should be covered in an intern curriculum include:
